# Addressing the Challenges to Diagnose Neonatal Hemolytic Anemia's Aetiologies in Low Ressources Countries: A Case Report

**DOI:** 10.1155/2020/3247127

**Published:** 2020-02-26

**Authors:** Lydie Ocini Ngolet, Josue Simo Louokdom, Nelly Guembo, Ocko Ngokaba Thibault

**Affiliations:** ^1^Department of Medical Hematology, Brazzaville Teaching Hospital, 13 Avenue Auxence Ikonga, BP 32, Brazzaville, Congo; ^2^Department of Pediatry, Brazzaville Blanche Gomes Hospital, Sassou Nguesso Boulevard, Brazzaville, Congo; ^3^Department of laboratory, Brazzaville Teaching Hospital, 13 Avenue Auxence Ikonga, BP 32, Brazzaville, Congo

## Abstract

*Background*. The diagnosis of neonatal hemolysis is an easy exercise. However, the diagnosis of its etiology can be very challenging especially in low ressources countries where laboratory capacities are limited. We report the case of hemolytic anemia episodes that started in the neonatal period, for which the trigger factor, infectious of paracetamol, is debatable.

## 1. Case Presentation

A 3-month-old and 2-day-old male infant was referred to the hematology department of the University Hospital for anemia.

The patient was the product on an uncomplicated full-term pregnancy. His medical history revealed neonatal jaundice at 5 days of life and 2 episodes of acute anemia following febrile runny nose, respectively, at 1 and 2 months. They required blood transfusion (80 mL each) and antipyretic (paracetamol 10 mg/Kg every 6 hours when needed) and empiric antibiotics for the second episode (amoxicillin 50 mg/kg every 12 hours during 5 days). The malaria smear was negative. The hemoglobin electrophoresis was also negative for the hemoglobin S. The rate of the hemoglobin F was 12%, which for the age of 1 monthwas normal.

Since the hemolytic anemia reoccurred 32 days after the last episode, the infant was referred to the hematology clinic at the University Hospital (June 12, 2019).

On physical examination, the patient was in good health, dynamic, and reactive. His rectal temperature was 36.6°C. There was pallor but no jaundice. The blood pressure was 129/88 mmHg and pulse rate was 120 beats per min, with enlarged spleen but no hepatomegaly. The rest of the physical was unremarkable.

After examination, we performed several blood tests (Table 1). The initial thin smear revealed at the microscopic inclusions in the red cells. Eleven percent of the patient's red cells were infected by parasites identified as *Plasmodium falciparum*. The malaria rapide test was positive for *Plasmodium falciparum*. We made the diagnosis of severe malaria based on the parasitemia and anemia. The mother tested also positive for *Plasmodium falciparum*. An antimalarial treatment with artesunate IV (3 mg/kg/j) was initiated for the mother and the baby for 3 days. The infant's smear was repeated after three days of antimalarial treatment. The results were still positive for *Plasmodium falciparum* (1% of the red cell infected). We decided to extend the antimalarial treatment for a total of 7 days. We requested additional laboratory studies to investigate the cause of the hemolytic anemia. The second hemoglobin electrophoresis was again negative for the hemoglobin S. The direct antiglobulin test was negative. The Glucose 6 Phosphate dehydrogenase level was low at 3.1 U/GHb (6.6 to 17.2). Table 1. We concluded that the patient was Glucose 6 Phosphate Dehydrogenase deficient (G6PD). The trigger factor of the hemolytic anemia was malaria.

At the follow-up visit (July 17, 2019), no improvement was noticed. The hemoglobin level was at 6.9 g/d, and the hemolysis was persistent (Table 1). The family denied any febrile episodes or drug intake. We reviewed carefully the environmental agents (as naphthalene ball) to which the infant was exposed and also the diet of the infant's mother and medication intake, since she was breastfeeding. The interview revealed that the mother had been self-medicating with paracetamol for a migraine condition since she was pregnant and she had presented two malaria episodes. We stopped the paracetamol and scheduled the infant for a follow-up.

The patient consulted 4 weeks and 3 days later. The hemoglobin rate was normal and, there were no biological signs of hemolysis.

## 2. Discussion

This case demonstrates the difficulty in diagnosing neonatal hemolytic anemia's etiology in Sub-Saharian Africa. The difficulties were related to not only the limit of diagnosis capacities but also the complexity of the case.

1. It was unclear to deduce if the patient developed congenital malaria or was infected in the postnatal period. Congenital malaria is defined as the presence of plasmodium asexual stages in newborn's cord or peripheral blood during the first week of life as a result of maternofetal transfer of parasites [1]. In areas endemic for malaria, symptomatic malaria infection is rare because of the effectiveness of the placenta as a barrier and the high level of maternal's antibodies and hemoglobin F [2, 3]. It is reported that it might take 3 to 4 weeks before congenitally-infected infants present symptoms [4]. Neonatal jaundice when related to malaria is usually associated with fever, irritability, and hepatosplenomegaly. Congenital malaria is characterized by low parasitemia, and the diagnosis is often missed because malaria smears, used in low ressources countries as a tool diagnosis, are often negative. The presence of *Plasmodium falciparum* in the blood of the mother and malaria history during the pregnancy concurred to the hypothesis of congenital malaria. The Polymerase Chain Reaction technique which is very sensitive in the detection of low parasite density, if available in the Congo, would have been helpful in the revision of our primary care but also our initial management. Repeated malaria smears turned out to be positive at the age of 3 months. The high density in *Plasmodium falciparum* may be the result of the progression of the disease or new contamination. Severe malaria is rare in the infant population and the population with G6PD deficiency [5]. An article from Mali examined the relationship between G6PD deficiency and severe malaria and reported that the G6PD deficiency confers highly significant protection against severe malaria in hemizygous males but not in heterozygous females [6].
2. The diagnosis of acquired hemolytic anemia triggered only by malaria was revised because the anemia was persistent despite the antimalarial treatment. The usual presentation of the X-linked disease is, in the Sub-Saharian African region, acute hemolytic anemia triggered by a factor. Between the crisis, the hemoglobin rate is usually back to normal [6]. The persistence of the anemia between crisis and absence of acute hemolytic anemia in the infant's male sibling contributed to delay in the diagnosis of G6PD deficiency. Since he got a femoral fracture during the delivery, the pediatrician assessed neonatal nonimmune hemolytic anemia due to trauma. The jaundice was related to the hematoma resorption. The repetition of the hemolysis episodes led to the diagnosis. A blood specimen sent to France helped in diagnosis of G6PD deficiency.
3. The nature of the triggered factor of the acute hemolysis anemia remains unclear. Despite the malaria treatment, the patient kept hemolyzing. Potential triggering factors were reviewed. There was no exposure to environmental factors as naphtalene balls or diet-related cause. The mother was exclusively breastfeeding. She reported paracetamol intake during her pregnancy. Paracetamol or acetaminophen is not an oxidant medication. It is not considered as a drug that can cause acute the hemolytic reaction. However, we found 2 case reports of hemolytic crisis following paracetamol ingestion through the database from Pubmed [7, 8]. Per the authors, the interindividual reaction to the drug intake in patients with G6PD deficient may explain that fact. The same agent may cause hemolysis to one patient and not to another one. Differences result not only from multiple factors such as genetics but also from maturation of liver enzymes [9]. It is not clear in our case to determine if the acute and prolonged anemia were associated with the mother's consumption of paracetamol. However, the hemolysis resolved since the mother stopped taking paracetamol.

At that stage, we can state the following:

- The anemia was hemolytic and regenerative, which excluded aplastic anemia.
- The hemolytic anemia was prolonged which is not common in G6PD deficiency. A G6PD genotype investigation, if available, would have been helpful.
- The hyperbilirubinemia initially hemolytic turned out to be progressively mixt without liver enzymes elevation which eliminated liver disease and conforted a chronic hemolytic anemia.
- The repeated hemoglobin electrophoresis was normal.

This observation highlights three points:

1. There is a need to build our diagnosis capacity but also to develop and implement a neonatal G6PD screening program in the Congo where the disease rate is theoretically high.
2. The need to establish guidelines for the diagnosis and management of neonatal jaundice and acute hemolytic anemia.
3. Congenital malaria should be suspected in all neonates in the Congo, endemic for malaria, who present with fever. Malaria smears and the rapid malaria test in the absence of the PCR technique should be repeated. Early diagnosis could prevent unnecessary antibiotic usage and avert the progression of malaria.

## 3. Conclusion

G6PD deficiency is a condition frequently underdiagnosed among infants in the Congo when symptoms are atypical. G6PD deficiency should be considered as a differential diagnosis in male infants with neonatal jaundice or hemolytic anemia.

## Figures and Tables

**Table 1:** Results of blood work.

Age Date	5 days 15-03-2019	1 month ½ 15-04-2019	2 months 14-05-2019	3 months 12-06-2019	4 months 17-07-2019	5 months 20-08-2019
Laboratory studies						
White cell count (G/L)	6.4	3.8	8.5	6.9	8.58	7.30
Differential count						
Neutrophils (%)	38	20	27.4	16.0	15.3	12.9
Lymphocytes (%)	54	70.8	60.9	73.5	76.9	61.6
Hemoglobin (g/dL)	9.6	5.0	8.6	7.9	6.9	12.5
Hematocrit (%)	28.9	15.0	28	25.9	27.5	30.7
Platelets (10^9^ g/L)	172	157	207	243	381	281
Reticulocyte count (G/L)	156.3	214	175.9	186.2	192.3	98.8
Serum bilirubin total (mg/L)	183.4			21.2	22	6.2
Serum bilirubin indirect (mg/L)	172.9			12.13	12.7	3.2
ALT (UI/L)					17	
AST (UI/L)					13	
Ferritin (*μ*g/L)				1518.4		390.4
Direct Coombs test		Negative		Negative		
Hemoglobin electrophoresis		A1 : 88% F.12%				A1 : 97.9% A2 : 2.1%
G6PD level (U/GHb)				3.1		
